# Women’s empowerment and health: nationwide insights on selected non-communicable conditions in Bangladesh

**DOI:** 10.1186/s12889-026-27084-y

**Published:** 2026-03-24

**Authors:** Tanjina Akter, Rafid Hassan, Md. Shahadoth Hossain, Sanjib Saha

**Affiliations:** 1 Department of Zoology, University of Dhaka, Dhaka, Bangladesh; 2 Nutrition Research Division, International Centre for Diarrhoeal Disease Research, Bangladesh (icddr,b), Dhaka, Bangladesh; 3 Department of Nutrition and Food Engineering, Daffodil International University, Dhaka, 1216 Bangladesh; 4 Health Economics Unit, Department of Clinical Science (Malmö), Lund University, Lund, Sweden

**Keywords:** Women’s empowerment, Survey-based women’s empowerment index (SWPER), Non-communicable diseases, Hypertension, Diabetes, Mental health, Anxiety, Depression, Demographic and health survey, Bangladesh.

## Abstract

**Background:**

Women’s empowerment may influence non-communicable disease (NCD) risk. However, evidence on these associations among women in Bangladesh remains limited. This study examined the associations between women’s empowerment and the prevalence of selected non-communicable conditions among ever-married reproductive-aged women in Bangladesh.

**Methods:**

Data from the 2022 Bangladesh Demographic and Health Survey were analyzed. Women’s empowerment was assessed using the survey-based women’s empowerment index (SWPER), encompassing three domains: attitudes toward violence, social independence, and decision-making. Selected non-communicable conditions included overweight/obesity, hypertension, diabetes, anxiety symptoms, and depression symptoms, and were categorized into composite physical NCDs (overweight/obesity, hypertension, or diabetes) and mental conditions (anxiety or depression symptoms). Anxiety and depression symptoms were screened using the GAD-7 and PHQ-9 scales. Both individual outcomes and composite outcomes were assessed. Modified Poisson regression was used to assess the associations between each empowerment domain and the different forms of NCDs while controlling for potential confounders and presented as adjusted prevalence ratio (APR).

**Results:**

The prevalence of overweight/obesity was 55.4%, hypertension 16.2%, diabetes 14.4%, anxiety 18.6%, and depression 4.7%. Overall, 63.2% of women had at least one physical NCD, and 19.4% had any mental symptoms. High empowerment was observed in the attitude toward violence (85.4%) and decision-making (59.5%) but was low in the social independence domain (16.9%). High empowerment in attitude toward violence was associated with lower prevalence of overweight/obesity (APR: 0.90, 95%CI: 0.82–1.00), any physical NCD (APR: 0.89, 95%CI: 0.79–1.00), anxiety symptoms (APR: 0.68, 95%CI: 0.58–0.79), depression symptoms (APR: 0.65, 95%CI: 0.45–0.94), and any mental symptoms (APR: 0.69, 95%CI: 0.59–0.80). Similarly, high empowerment in social independence was inversely associated with overweight/obesity (APR: 0.94, 95%CI: 0.88–1.00), anxiety symptoms (APR: 0.87, 95%CI: 0.78–0.97), and any mental symptoms (APR: 0.88, 95%CI: 0.80–0.98). However, higher decision-making empowerment was associated with higher prevalence of overweight/obesity (APR: 1.24, 95%CI: 1.15–1.33), hypertension (APR: 1.29, 95%CI: 1.01–1.64), and any physical NCD (APR: 1.12, 95%CI: 1.03–1.21).

**Conclusions:**

Women’s empowerment domains exhibited associations with selected health outcomes. Promoting gender-equitable attitudes and targeted interventions addressing social independence might improve the health and well-being of women in Bangladesh.

**Supplementary Information:**

The online version contains supplementary material available at 10.1186/s12889-026-27084-y.

## Background

Non-communicable diseases (NCDs) remain a major global health challenge, responsible for an estimated 43.8 million deaths and 1.73 billion disability-adjusted life years (DALYs) in 2021 [1]. Nearly two-thirds of these deaths arise in low-and middle-income countries (LMICs) such as Bangladesh, where premature mortality (before age 70) from NCDs represents about 82% of all early deaths [2]. Physical NCDs such as cardiovascular diseases, cancers, chronic respiratory diseases, and diabetes continue to dominate NCD-related mortality globally [2]. However, increasing attention is now being given to mental health disorders, particularly anxiety and depression, which affect more than 1.1 billion people [3]. Mental disorders rank among the top ten contributors to the global disease burden in terms of prevalence and DALYs, with a rising trend since 1990 [4]. Recognizing their importance, the 2018 United Nations Political Declaration on the Prevention and Control of NCDs officially broadened the NCDs agenda to include the promotion of mental health and well-being alongside the traditional NCDs [5]. Moreover, individuals with mental health conditions experience a disproportionately higher burden of physical NCDs, particularly in South Asia [6]. Notably, gender disparities are evident: women globally bear a greater burden of both mental disorders [4] and physical NCDs [1].

Bangladesh is undergoing a rapid epidemiological transition, with NCDs now accounting for approximately 70% of all deaths [7]. In 2019, five of the top ten risk factors for all-cause mortality and DALYs were physical NCD-related metabolic factors, including high blood pressure, high fasting plasma glucose, elevated cholesterol, and high body mass index (BMI)—conditions that contribute to hypertension, diabetes, and overweight/obesity [8]. Mental health problems are also an increasing concern here. Women, in particular, experience a more complex health trajectory across their life course due to both physiological and socioeconomic factors, which exacerbate their vulnerability to physical NCDs and mental disorders. Evidence suggests that Bangladeshi women face higher rates of these conditions compared to men [9, 10]. In 2022, 38% of women were overweight/obese, 23% had hypertension, 17% had diabetes, 5% experienced depression, and 20% reported anxiety [10].

Women’s empowerment is intrinsically linked to improved health outcomes and is enshrined in the Sustainable Development Goals (SDGs), particularly Goal 5, which seeks to “empower all women and girls” [11]. Empowerment encompasses multidimensional processes that enhance an individual’s capacity to make choices and take actions that positively influence their health and well-being [12]. Empowered women generally enjoy increased access to resources, greater decision-making authority, and enhanced agency, all of which are associated with better health behaviors and more effective engagement with preventive and curative health services [13]. Evidence consistently demonstrates that higher levels of empowerment yield substantial benefits for reproductive, maternal, newborn, and child health, including increased use of modern contraception, improved maternal care uptake, and better child nutrition and immunization coverage [14–19].

Despite growing recognition of the pivotal role of women’s empowerment in shaping health trajectories, empirical data on the association between empowerment and NCD risk among Bangladeshi women remain scarce. The challenge of rigorously measuring empowerment—given its abstract and multidimensional nature—has led to diverse frameworks and tools, limiting comparability across studies. The survey-based women’s empowerment index (SWPER), developed and validated using demographic and health survey data, overcomes many of these limitations by capturing key domains of empowerment (attitude toward violence, social independence, and decision-making) using robust, individual-level measures [20].

This study addresses critical knowledge gaps by examining the association between women’s empowerment and selected physical and mental non-communicable conditions among women of reproductive age (WRA) in Bangladesh, using the latest nationally representative data. Findings from this research are expected to offer insights that inform transformative approaches for reducing the NCDs burden and improving women’s health in Bangladesh and similar LMICs settings.

## Methods

### Data source and study design

This study analyzed secondary data from the 2022 Bangladesh demographic and health survey (BDHS). This cross-sectional survey followed a two-stage, stratified sampling for enumeration areas (EAs) selection covering both rural and urban areas from eight administrative divisions. Firstly, probability proportional to size sampling was employed to select 675 EAs (urban areas: 237, rural areas: 438). Subsequently, from each EA using systematic sampling, 45 households were selected, among which 30 households with ever-married women aged 15–49 years were randomly chosen for a detailed interview. For biomarker measurements, a subsample of households was selected systematically, with half of the surveyed households included for anthropometric assessment. From this subsample, eight households were further selected for additional biomarker measurements, including anthropometry, blood pressure (BP), and blood glucose assessments.

**Fig. 1:**
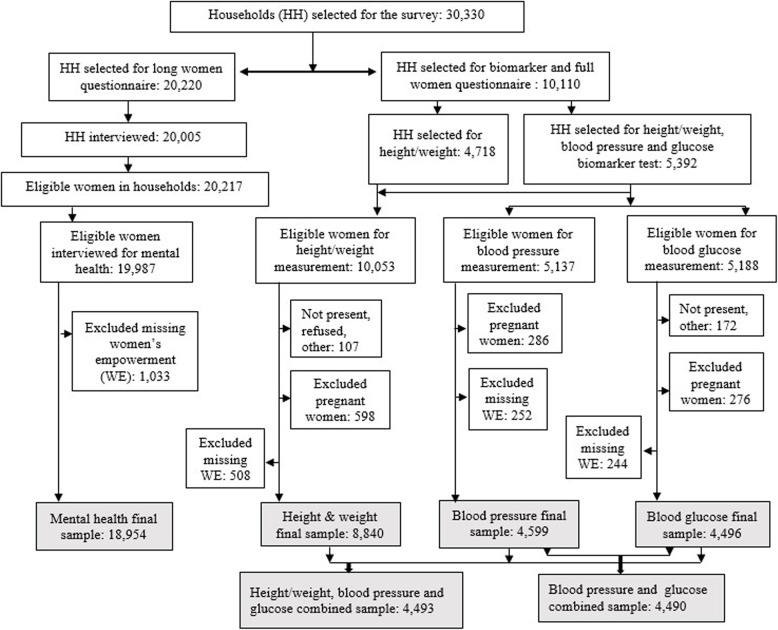
Schematic diagram of sample selection

A total of 19,987 eligible WRA were interviewed using the long questionnaire. Among them, 10,053 WRA were eligible for biomarker measurements (10,053 for height and weight, 5,137 for blood pressure, and 5,188 for blood glucose testing). In this study, pregnant women and participants with missing information on biomarkers or women’s empowerment variables were excluded. The final analytical samples included 18,954 women for mental health analysis, 8,840 for anthropometric outcomes, 4,599 for blood pressure, and 4,496 for blood glucose analysis. In addition, 4,493 women had complete data for all three biomarkers (Fig. 1).

BP and fasting blood glucose were measured by trained health technicians following standardized protocols. BP was recorded three times at intervals of at least five minutes using the Multi-User Upper Arm Blood Pressure Monitor (model UA-767 F/FAC), and the mean of the second and third readings was considered. Fasting blood glucose was assessed using a HemoCue 201 RT analyzer. Capillary blood samples were obtained from the middle or ring finger after an overnight fast, with the first two drops discarded and the third used for analysis. Anthropometric measurements were taken using calibrated equipment: weight was measured with a SECA digital scale (model 874U), and height was recorded using a ShorrBoard measuring board.

Anxiety was evaluated using the Generalized Anxiety Disorder-7 (GAD-7) scale [21]. Participants indicated the frequency of their symptoms on a scale from 0 (never) to 3 (always) over the past two weeks, with responses to the seven items combined to provide a total score ranging from 0 to 21. The GAD-7 showed a strong internal consistency (Cronbach’s α = 0.827). Depression was evaluated using the Patient Health Questionnaire-9 (PHQ-9) scale [22]. Participants indicated the frequency of their symptoms on a scale from 0 (never) to 3 (always), with responses to the 9 items combined to provide a total score ranging from 0 to 27. The PHQ-9 scale also showed a strong internal consistency (Cronbach’s α = 0.815). Detailed descriptions of the sampling design and data collection are available in the report [10].

### Outcome variables

The primary outcome of this study was the presence of selected non-communicable conditions, which were further classified into physical and mental NCDs based on their availability in the BDHS dataset. In this study, physical NCDs included overweight/obesity, hypertension, and diabetes, whereas mental health conditions included anxiety and depression symptoms. Both individual and composite outcomes were assessed.

Women were classified as overweight/obese if their BMI was ≥ 23 kg/m², following the Asian cut-off for BMI [23]. Hypertension was defined according to World Health Organization (WHO) criteria as an average systolic blood pressure ≥ 140 mmHg, an average diastolic blood pressure ≥ 90 mmHg, or current use of antihypertensive medication [24]. Similarly, diabetes was defined based on WHO guidelines as a fasting blood glucose level ≥ 7 mmol/L or current use of glucose-lowering medication [25]. Different studies have used varying cut points to determine anxiety symptoms. In this study, a GAD-7 score ≥ 6 was used to identify anxiety symptoms to align with the BDHS report [10, 21]. We also conducted sensitivity analysis using another established cut point (GAD-7 scores ≥ 10) of moderate/severe anxiety symptoms [21]. Furthermore, depression symptoms were defined as a PHQ-9 score ≥ 10 [10, 22].

Two composite outcomes were constructed. Any physical NCD was defined as the presence of overweight/obesity, hypertension, or diabetes [26, 27]. Any mental health symptom was defined as having either anxiety or depression symptoms.

### Exposure variables

The primary exposure variable in this study was women’s empowerment, assessed using the SWPER Global Index. This standardized and validated index, developed from DHS data, measures women’s empowerment across LMICs through three different domains: attitude toward violence, social independence, and decision-making [20]. The attitude toward violence dimension was constructed from five DHS items assessing whether a woman believes wife beating is justified under specific circumstances, such as going out without informing the husband, arguing with him, refusing sex, neglecting children, or burning food. The social independence domain incorporates indicators such as the frequency of reading newspapers or magazines, years of schooling, age at first cohabitation/marriage, age at first birth, as well as age and educational differences between the woman and her husband. The decision-making domain includes women’s participation in household decisions and was based on three questions concerning who usually decides about the woman’s healthcare, major household purchases, and visits to family or relatives.

Following the recommended SWPER Global methodology, the index was constructed using the published principal component analysis (PCA) loadings and item weights [20]. Domain-specific scores were calculated by applying these predefined weights to the corresponding DHS items. To ensure global comparability, the resulting scores were standardized using the global mean and standard deviation derived from previous studies of LMICs [20], according to the formula:

$$\:Standardized\:score=\frac{\mathrm{R}\mathrm{a}\mathrm{w}\:\mathrm{s}\mathrm{c}\mathrm{o}\mathrm{r}\mathrm{e}-\mathrm{G}\mathrm{l}\mathrm{o}\mathrm{b}\mathrm{a}\mathrm{l}\:\mathrm{m}\mathrm{e}\mathrm{a}\mathrm{n}}{\mathrm{G}\mathrm{l}\mathrm{o}\mathrm{b}\mathrm{a}\mathrm{l}\:\mathrm{S}\mathrm{D}}$$

These standardized scores, which range from negative to positive values, indicate levels of empowerment, with positive values representing above-average empowerment, negative values indicating below-average empowerment, and zero representing the global average. Subsequently, these scores were classified into three categories: low, medium, and high empowerment, following previous methodology [20].

### Covariates

Covariate selection was guided by their association with both outcome and exposure variables in the prior literature [28–35]. The following covariates were included: women age in years (15–29, 30–39, 40–49), women education (no education, primary, secondary, higher), women employment status (unemployed, employed), number of children (up to 2, 3 or more), area of residence (urban, rural), and administrative region (Barisal, Chattogram, Dhaka, Khulna, Mymensingh, Rajshahi, Sylhet). The household wealth index was constructed using PCA of the key assets owned by households [10]. Households were further ranked based on wealth scores and divided into quintiles (poorest, poorer, middle, richer, richest).

### Statistical analyses

Descriptive analyses were performed to summarize the study variables. Proportions were determined for categorical variables, while means were presented for continuous variables. Bivariate associations between the outcome variables and sociodemographic as well as empowerment characteristics were examined using chi-square tests of independence. To explore the association of each of the empowerment domains and non-communicable conditions, modified Poisson regression with robust variance procedure to estimate standard errors was used [36–38]. Adjusted prevalence ratio (APR) with corresponding 95% confidence intervals (CIs) was estimated, controlling for all covariates, including women’s age, employment status, number of children, wealth index, area of residence, and administrative division, simultaneously. However, education was not adjusted in the regression models to prevent over-adjustment, as it was an intrinsic component of the SWPER domain. Before running the models, multicollinearity was assessed using variance inflation factors (VIF). The observed VIF values ranged from 1.01 to 2.20, all of which were below the threshold of 10 [39]. All statistical tests were two-tailed, and a p-value < 0.05 was considered statistically significant. “svy” command was used to account for clustering, stratification, and sampling weights in all analyses. Statistical analyses were conducted using Stata version 15.0 (StataCorp LLC, College Station, TX, USA).

## Results

This study included a total of 8,840 women for overweight/obesity, 4,599 for hypertension, 4,496 for diabetes, and 18,954 for mental illness. Most of the women were aged 30–39 years. Regarding educational attainment, over half of the participants had secondary or higher education, while over one in ten had no formal education. Around two-thirds of women were unemployed and had up to two children. Most women resided in rural areas. Geographically, participants were spread across all divisions, with the highest proportion from Dhaka and Chattogram. The SWPER standardized score indicated relatively higher empowerment in the “attitude toward violence” 0.55 and “decision-making” domain 0.36 to 0.38 compared to “social independence” −0.35 to − 0.38 (Table 1). Furthermore, participants’ characteristics for hypertension/diabetes and any physical NCDs are presented in Supplementary Table 1.

**Table 1:** Sociodemographic characteristics of the studied participants

Variables	Overweight/obesity, *N* = 8,840	Hypertension, *N* = 4,599	Diabetes, *N* = 4,496	Anxiety/Depression, *N* = 18,954
	*n* (%)	*n* (%)	*n* (%)	*n* (%)
Women age in years				
15–29	3550 (40.6)	1744 (37.8)	1707 (37.8)	8172 (43.7)
30–39	3133 (35.5)	1666 (36.7)	1621 (36.5)	6475 (34.0)
40–49	2157 (23.9)	1189 (25.5)	1168 (25.7)	4307 (22.3)
Women education				
No education	1172 (13.3)	648 (14.2)	634 (14.2)	2431 (13.0)
Primary	2304 (26.0)	1231 (27.0)	1206 (27.0)	4886 (25.8)
Secondary	4040 (47.1)	2023 (45.2)	1982 (45.4)	8786 (47.4)
Higher	1324 (13.6)	697 (13.6)	674 (13.4)	2851 (13.8)
Women employment status				
Not employed	6047 (67.0)	3134 (66.4)	3062 (66.3)	13,254 (68.8)
Employed	2793 (33.0)	1465 (33.6)	1434 (33.7)	5700 (31.2)
Number of children				
Up to 2	5967 (67.2)	3007 (64.8)	2937 (64.8)	13,173 (69.4)
3 or more	2,873 (32.8)	1,592 (35.2)	1559 (35.2)	5781 (30.6)
Wealth index				
Poorest	1583 (17.7)	804 (17.4)	797 (17.7)	3350 (17.6)
Poorer	1674 (19.6)	890 (20.1)	881 (20.5)	3721 (20.2)
Middle	1794 (20.9)	892 (20.0)	876 (20.0)	3777 (20.6)
Richer	1833 (21.0)	958 (21.0)	939 (21.0)	3936 (21.0)
Richest	1956 (20.8)	1055 (21.5)	1003 (20.8)	4170 (20.6)
Area of residence				
Urban	3087 (28.3)	1620 (28.5)	1568 (28.0)	6611 (28.2)
Rural	5753 (71.7)	2979 (71.5)	2928 (72.0)	12,343 (71.8)
Division				
Barishal	957 (6.2)	524 (6.5)	516 (6.6)	2030 (6.0)
Chattogram	1279 (18.1)	689 (19.0)	672 (19.0)	2811 (18.7)
Dhaka	1322 (25.2)	663 (24.4)	619 (23.6)	2866 (25.3)
Khulna	1129 (11.7)	592 (12.0)	580 (12.0)	2474 (12.0)
Mymensingh	971 (7.8)	499 (7.8)	495 (8.0)	2049 (7.6)
Rajshahi	1169 (13.6)	591 (13.2)	583 (13.4)	2436 (13.2)
Rangpur	1094 (11.8)	551 (11.3)	548 (11.5)	2294 (11.5)
Sylhet	919 (5.6)	490 (5.8)	483 (5.9)	1994 (5.7)
Standardized attitude toward violence SWPER score, mean (95%CI)	0.55 (0.53, 0.56)	0.55 (0.53, 0.56)	0.55 (0.53, 0.56)	0.55 (0.54, 0.56)
Standardized social independence SWPER score, mean (95%CI)	−0.37 (− 0.39, − 0.34)	−0.38 (− 0.41, − 0.35)	−0.38 (− 0.41, − 0.35)	−0.35 (− 0.37, − 0.33)
Standardized decision-making SWPER score, mean (95%CI)	0.37 (0.34, 0.39)	0.38 (0.35, 0.41)	0.38 (0.35, 0.41)	0.36 (0.34, 0.38)

Overall, most women (85.4%) reported high empowerment in the attitude toward violence domain, followed by the decision-making domain (59.5%), while high empowerment was much lower in the social independence domain (16.9%). Empowerment patterns varied across sociodemographic groups: younger women were more empowered in attitude toward violence, while older women showed greater empowerment in decision-making. Higher levels of education corresponded with greater proportions of women reporting high empowerment across all domains. Moreover, wealthier and urban women exhibited higher levels of empowerment, and notable regional variations were observed (Table 2).

**Table 2:** Level of women's empowerment by sociodemographic characteristics (*N* = 18,954**)**

Variables	Level of empowerment^a^											
	Attitude toward violence				Social independence				Decision-making			
	Low	Medium	High	*p*-value	Low	Medium	High	*p*-value	Low	Medium	High	*p*-value
Overall	678 (3.9)	1932 (10.7)	16,344 (85.4)	-	7996 (43.3)	7509 (39.8)	3449 (16.9)	-	2827 (14.1)	5048 (26.4)	11,079 (59.5)	-
Women age in years												
15–29	254 (3.5)	746 (9.5)	7172 (87.0)	< 0.001	2849 (35.8)	3856 (47.1)	1477 (17.1)	< 0.001	1631 (18.8)	2424 (29.5)	4117 (51.7)	< 0.001
30–39	236 (3.9)	696 (11.4)	5543 (84.7)		2713 (43.1)	2451 (38.3)	1311 (18.6)		703 (10.3)	1597 (24.3)	4175 (65.4)	
40–49	188 (4.6)	490 (12.0)	3629 (83.4)		2444 (58.3)	1202 (27.6)	661 (14.1)		493 (10.9)	1027 (23.5)	2787 (65.6)	
Women education												
No education	142 (6.2)	310 (13.1)	1979 (80.7)	< 0.001	1971 (81.7)	375 (14.9)	85 (3.4)	< 0.001	363 (14.2)	610 (24.6)	1458 (61.2)	< 0.001
Primary	236 (4.9)	573 (12.3)	4077 (82.8)		3090 (64.1)	1539 (30.8)	257 (5.1)		731 (14.0)	1252 (24.9)	2903 (61.1)	
Secondary	271 (3.4)	874 (10.5)	7641 (86.1)		2886 (33.6)	4698 (53.4)	1202 (13.0)		1398 (15.1)	2420 (27.3)	4968 (57.6)	
Higher	29 (1.3)	175 (6.1)	2647 (92.6)		49 (1.7)	897 (33.1)	1905 (65.2)		335 (11.0)	766 (27.9)	1750 (61.1)	
Women employment status												
Not employed	451 (3.6)	1299 (10.3)	11,504 (86.1)	0.004	5162 (40.0)	5587 (42.4)	2505 (17.6)	< 0.001	2288 (16.4)	3677 (27.3)	7289 (56.3)	< 0.001
Employed	227 (4.3)	633 (11.7)	4840 (84.0)		2834 (50.4)	1922 (34.1)	944 (15.5)		539 (9.2)	1371 (24.3)	3790 (66.5)	
Number of children												
Up to 2	418 (3.5)	1268 (10.1)	11,487 (86.4)	< 0.001	4435 (34.9)	5677 (43.5)	3061 (21.6)	< 0.001	2101 (15.2)	3656 (27.6)	7416 (57.2)	< 0.001
3 or more	260 (4.7)	664 (12.1)	4857 (83.2)		3561 (62.3)	1832 (31.2)	388 (6.5)		726 (11.7)	1392 (23.6)	3663 (64.7)	
Wealth index												
Poorest	181 (5.5)	391 (11.9)	2778 (82.6)	< 0.001	1820 (54.9)	1256 (37.2)	274 (7.9)	< 0.001	551 (16.0)	911 (26.4)	1888 (57.6)	< 0.001
Poorer	162 (4.8)	433 (11.9)	3126 (83.3)		1817 (49.3)	1468 (39.2)	436 (11.5)		600 (15.7)	994 (26.1)	2127 (58.2)	
Middle	130 (3.8)	414 (11.4)	3233 (84.8)		1680 (45.0)	1557 (41.1)	540 (13.9)		599 (14.6)	970 (25.8)	2208 (59.6)	
Richer	127 (3.4)	380 (10.3)	3429 (86.3)		1590 (41.9)	1621 (40.9)	725 (17.2)		591 (13.8)	1041 (25.7)	2304 (60.5)	
Richest	78 (2.1)	314 (8.1)	3778 (89.8)		1089 (27.2)	1607 (40.0)	1474 (32.8)		486 (11.0)	1132 (27.9)	2552 (61.1)	
Area of residence												
Urban	157 (2.7)	601 (9.8)	5853 (87.5)	0.004	2375 (36.7)	2559 (38.6)	1677 (24.7)	< 0.001	850 (11.1)	1758 (26.1)	4003 (62.8)	< 0.001
Rural	521 (4.3)	1331 (11.1)	10,491 (84.6)		5621 (45.9)	4950 (40.2)	1772 (13.9)		1977 (15.3)	3290 (26.5)	7076 (58.2)	
Division												
Barishal	50 (2.8)	187 (9.2)	1793 (88.0)	0.004	817 (42.0)	837 (42.3)	376 (15.7)	< 0.001	352 (18.5)	639 (31.6)	1039 (49.9)	< 0.001
Chattogram	89 (3.3)	274 (9.9)	2448 (86.8)		979 (35.6)	1320 (47.7)	512 (16.7)		347 (13.4)	646 (22.1)	1818 (64.5)	
Dhaka	119 (4.1)	347 (11.5)	2400 (84.4)		1158 (40.0)	1119 (39.2)	589 (20.8)		335 (11.2)	849 (29.7)	1682 (59.1)	
Khulna	118 (4.7)	294 (12.1)	2062 (83.2)		1196 (50.1)	885 (35.4)	393 (14.5)		457 (19.1)	651 (25.8)	1366 (55.1)	
Mymensingh	80 (4.0)	177 (8.8)	1792 (87.2)		888 (44.3)	819 (40.1)	342 (15.6)		320 (15.5)	468 (22.7)	1261 (61.8)	
Rajshahi	94 (4.5)	295 (13.0)	2047 (82.5)		1208 (52.1)	850 (34.6)	378 (13.3)		303 (11.9)	684 (27.0)	1449 (61.1)	
Rangpur	61 (3.0)	214 (9.8)	2019 (87.2)		1143 (51.7)	801 (35.0)	350 (13.3)		270 (11.9)	552 (24.5)	1472 (63.6)	
Sylhet	67 (3.7)	144 (7.4)	1783 (88.9)		607 (31.3)	878 (44.1)	509 (24.6)		443 (23.2)	559 (28.4)	992 (48.4)	

^a^Values were reported as number (%)

Over half of the participants (55.4%) were overweight/obese, while 16.2% had hypertension and 14.4% had diabetes. Anxiety and depression affected 18.6% and 4.7% of participants, respectively. Overall, 19.4% of women had at least one mental symptom, whereas 63.2% had at least one physical NCD (Table 3). Besides, the prevalence of anxiety symptoms using the GAD-7 ≥ 10 cut point, and hypertension/diabetes are presented in Supplementary Table 2.

**Table 3:** Prevalence of physical, and mental NCDs by sociodemographic factors and SWPER domains

Variables	Individual outcomes										Composite outcomes			
	Overweight/obesity, *N* = 8840		Hypertension, *N* = 4599		Diabetes, *N* = 4496		Anxiety symptoms, *N* = 18,954		Depression symptoms, *N* = 18,954		Any mental symptoms, *N* = 18,954		Any physical NCDs, *N* = 4490	
	*n* (%)	*p*-value	*n* (%)	*p*-value	*n* (%)	*p*-value	*n* (%)	*p*-value	*n* (%)	*p*-value	*n* (%)	*p*-value	*n* (%)	*p*-value
Overall	4906 (55.4)	-	773 (16.2)	-	650 (14.4)	-	3550 (18.6)	-	912 (4.7)	-	3695 (19.4)	-	2845 (63.2)	-
Women age in years														
15–29	1568 (44.0)	< 0.001	96 (5.0)	< 0.001	142 (8.7)	< 0.001	1169 (14.0)	< 0.001	318 (3.7)	< 0.001	1232 (14.8)	< 0.001	869 (51.0)	< 0.001
30–39	1968 (63.0)		301 (17.3)		230 (14.2)		1375 (21.5)		329 (5.0)		1416 (22.1)		1110 (68.1)	
40–49	1370 (63.4)		376 (31.1)		278 (23.1)		1006 (23.3)		265 (5.9)		1047 (24.2)		866 (74.4)	
Women education														
No education	581 (49.4)	< 0.001	148 (22.5)	< 0.001	101 (17.0)	0.135	629 (25.8)	< 0.001	166 (6.7)	< 0.001	651 (26.8)	< 0.001	389 (62.3)	0.294
Primary	1214 (54.4)		226 (18.3)		189 (15.5)		1043 (21.2)		258 (5.0)		1074 (21.9)		737 (62.8)	
Secondary	2268 (55.6)		304 (14.1)		265 (13.1)		1515 (17.2)		390 (4.4)		1575 (17.8)		1253 (62.7)	
Higher	843 (62.3)		95 (12.4)		95 (13.9)		363 (12.2)		98 (2.9)		395 (13.2)		466 (67.0)	
Women employment status														
Not employed	3350 (55.2)	0.698	530 (16.0)	0.681	459 (15.1)	0.099	2415 (18.3)	0.242	630 (4.6)	0.744	2523 (19.1)	0.347	1928 (63.3)	0.966
Employed	1556 (55.7)		243 (16.5)		191 (13.0)		1135 (19.3)		282 (4.7)		1172 (19.9)		917 (63.2)	
Number of children														
Up to 2	3268 (53.9)	< 0.001	451 (14.6)	< 0.001	403 (13.9)	0.267	2179 (16.3)	< 0.001	571 (4.1)	< 0.001	2286 (17.1)	< 0.001	1833 (61.8)	0.017
3 or more	1638 (58.4)		322 (19.1)		247 (15.3)		1371 (23.8)		341 (5.8)		1409 (24.4)		1012 (65.9)	
Wealth index														
Poorest	592 (37.6)	< 0.001	110 (13.8)	0.007	67 (7.8)	< 0.001	700 (20.9)	< 0.001	179 (5.1)	0.071	724 (21.6)	< 0.001	369 (46.2)	< 0.001
Poorer	814 (49.8)		136 (14.2)		110 (12.3)		790 (21.1)		188 (5.0)		817 (21.9)		497 (57.3)	
Middle	950 (53.4)		137 (14.9)		120 (13.0)		699 (18.4)		200 (5.2)		729 (19.2)		542 (63.0)	
Richer	1126 (61.1)		181 (18.6)		141 (15.2)		730 (18.1)		186 (4.4)		762 (18.8)		657 (69.7)	
Richest	1424 (71.9)		209 (18.8)		212 (22.7)		631 (15.1)		159 (3.6)		663 (15.9)		780 (77.3)	
Area of residence														
Urban	1970 (63.6)	< 0.001	320 (19.1)	0.001	294 (20.3)	< 0.001	1181 (16.9)	0.024	297 (4.0)	0.126	1235 (17.6)	0.024	1107 (70.9)	< 0.001
Rural	2936 (52.1)		453 (15.0)		356 (12.1)		2369 (16.3)		615 (4.9)		2460 (20.1)		1738 (60.3)	
Division														
Barishal	555 (54.9)	< 0.001	74 (13.8)	0.178	72 (14.3)	< 0.001	358 (17.9)	< 0.001	87 (4.5)	< 0.001	385 (19.2)	< 0.001	336 (62.6)	< 0.001
Chattogram	785 (59.9)		124 (17.1)		100 (14.7)		571 (20.4)		113 (4.0)		587 (21.0)		465 (68.4)	
Dhaka	770 (59.0)		94 (14.8)		126 (20.7)		458 (15.9)		120 (3.9)		479 (16.6)		401 (65.7)	
Khulna	695 (60.0)		104 (17.0)		77 (11.7)		491 (19.1)		162 (6.2)		511 (19.8)		394 (66.2)	
Mymensingh	418 (41.9)		79 (15.3)		56 (10.8)		329 (16.6)		70 (3.5)		348 (17.4)		256 (50.7)	
Rajshahi	685 (56.3)		108 (18.2)		64 (8.2)		415 (17.0)		83 (3.4)		434 (17.7)		383 (63.3)	
Rangpur	561 (49.0)		88 (14.2)		77 (13.2)		543 (24.8)		158 (7.3)		561 (25.7)		333 (59.1)	
Sylhet	437 (45.5)		102 (20.5)		78 (15.5)		385 (18.9)		119 (5.9)		390 (19.1)		277 (55.8)	
Attitude toward violence														
Low	181 (58.7)	0.509	36 (18.4)	0.402	23 (12.9)	0.584	192 (27.4)	< 0.001	49 (7.0)	0.001	196 (27.9)	< 0.001	114 (68.9)	0.162
Medium	497 (55.2)		86 (18.1)		70 (16.0)		456 (22.8)		123 (6.1)		478 (24.2)		295 (66)	
High	4228 (55.3)		651 (15.9)		557 (14.3)		2902 (17.7)		740 (4.4)		3021 (18.4)		2436 (62.6)	
Social independence														
Low	2069 (55.4)	0.101	373 (18.2)	0.011	277 (14.6)	0.193	1691 (20.7)	< 0.001	420 (5.1)	0.018	1749 (21.5)	< 0.001	1219 (64.3)	0.019
Medium	1889 (54.2)		264 (14.4)		241 (13.3)		1318 (17.8)		351 (4.5)		1371 (18.4)		1075 (60.7)	
High	948 (58.1)		136 (14.9)		132 (16.5)		541 (15.4)		141 (3.7)		575 (16.3)		551 (66.5)	
Decision-making														
Low	572 (42.6)	< 0.001	86 (11.6)	< 0.001	82 (10.9)	0.028	448 (16.1)	0.007	111 (4.1)	0.300	469 (17.0)	0.016	372 (54.2)	< 0.001
Medium	1295 (55.2)		183 (14.8)		179 (15.8)		934 (18.2)		220 (4.5)		978 (19.0)		731 (62.8)	
High	3039 (58.4)		504 (17.8)		389 (14.7)		2168 (19.4)		581 (4.9)		2248 (20.1)		1742 (65.4)	

The prevalence of all individual and composite physical NCDs increased significantly with age, greater household wealth, urban residence, and higher levels of empowerment in decision-making domains. Women with higher education, greater parity, and higher empowerment in social independence had a lower prevalence of hypertension. Furthermore, all forms of mental NCDs were more prevalent among older women, those with lower education, higher parity, and lower empowerment regarding attitude toward violence and social independence. Women living in urban areas, from poorer households, and with higher decision-making empowerment had a higher prevalence of anxiety. Furthermore, a significant regional disparity in the prevalence of all forms of NCDs was observed (Table 3).

Supplementary Tables 3–4 present the unadjusted prevalence ratio for the associations of women’s empowerment domains and different forms of NCD outcomes. When examining individual physical NCD outcomes, in the adjusted model, women with high empowerment in the attitude toward violence (APR: 0.90, 95%CI: 0.82–1.00, *p* = 0.047) and social independence domain (APR: 0.94, 95%CI: 0.88–1.00, *p* = 0.046) had significantly lower prevalence of overweight/obesity. However, high empowerment in the decision-making domain was significantly associated with higher prevalence of overweight/obesity (APR: 1.24, 95%CI: 1.15–1.33, *p* < 0.001) and hypertension (APR: 1.29, 95%CI: 1.01–1.64, *p* = 0.042). In terms of composite outcome, any physical NCDs had a significant inverse association with a higher level of empowerment in the attitude toward violence domain (APR: 0.89, 95%CI: 0.79–1.00, *p* = 0.048) and a positive association with a higher level of empowerment in the decision-making domain (APR: 1.12, 95%CI: 1.03–1.21, *p* = 0.008) (Table 4). Furthermore, women with medium empowerment in the decision-making domain had significantly higher prevalence of diabetes/hypertension (APR: 1.21, 95%CI: 1.01–1.46, *p* = 0.045) (Supplementary Table 5).

**Table 4:** Associations between women’s empowerment domains and different forms of physical NCDs

Variables	Overweight/obesity		Hypertension		Diabetes		Any physical NCDs	
	APR (95%CI)	*p*-value	APR (95%CI)	*p*-value	APR (95%CI)	*p*-value	APR (95%CI)	*p*-value
Attitude toward violence								
Low	Ref.		Ref.		Ref.		Ref.	
Medium	0.92 (0.82, 1.03)	0.152	1.00 (0.69, 1.46)	0.995	1.17 (0.73, 1.86)	0.515	0.95 (0.83, 1.09)	0.495
High	0.90 (0.82, 1.00)	0.047	0.90 (0.65, 1.26)	0.543	1.00 (0.65, 1.52)	0.985	0.89 (0.79, 1.00)	0.048
Social independence								
Low	Ref.		Ref.		Ref.		Ref.	
Medium	0.98 (0.94, 1.03)	0.387	0.93 (0.79, 1.08)	0.340	0.92 (0.77, 1.11)	0.390	0.95 (0.90, 1.01)	0.086
High	0.94 (0.88, 1.00)	0.046	0.82 (0.66, 1.01)	0.058	0.91 (0.72, 1.17)	0.467	0.96 (0.90, 1.03)	0.275
Decision-making								
Low	Ref.		Ref.		Ref.		Ref.	
Medium	1.21 (1.12, 1.30)	< 0.001	1.19 (0.92, 1.55)	0.190	1.30 (1.00, 1.70)	0.052	1.10 (1.00, 1.20)	0.043
High	1.24 (1.15, 1.33)	< 0.001	1.29 (1.01, 1.64)	0.042	1.14 (0.90, 1.46)	0.281	1.12 (1.03, 1.21)	0.008

All models were adjusted for women age, employment status, number of children, wealth index, area of residence, and division

*APR* Adjusted prevalence ratio, *CI* Confidence interval, *Ref* Reference

For mental health outcomes, higher empowerment in the attitude toward violence domain was significantly associated with a lower prevalence of anxiety symptoms (APR: 0.68, 95%CI: 0.58–0.79, *p* < 0.001), depression symptoms (APR: 0.65, 95%CI: 0.45–0.94, *p* = 0.021), and any mental symptoms (APR: 0.69, 95%CI: 0.59–0.80, *p* < 0.001). Similarly, high social independence empowerment showed significantly inverse associations with anxiety symptoms (APR: 0.87, 95%CI: 0.78–0.97, *p* = 0.011) and any mental symptoms (APR: 0.88, 95%CI: 0.80–0.98, *p* = 0.016). However, decision making domain had no significant association with any of the mental symptoms (Table 5). Besides, when applying the alternative cut-off for anxiety (GAD-7 ≥ 10), only high empowerment in the attitude toward violence domain remained inversely associated with anxiety symptoms (APR: 0.60, 95%CI: 0.43–0.84, *p* = 0.003) (Supplementary Table 6).

**Table 5:** Associations between women’s empowerment domains and different forms of mental symptoms

Variables	Anxiety symptoms		Depression symptoms		Any mental symptoms	
	APR (95%CI)	*p*-value	APR (95%CI)	*p*-value	APR (95%CI)	*p*-value
Attitude toward violence						
Low	Ref.		Ref.		Ref.	
Medium	0.84 (0.71, 1.00)	0.044	0.87 (0.57, 1.32)	0.517	0.87 (0.73, 1.03)	0.110
High	0.68 (0.58, 0.79)	< 0.001	0.65 (0.45, 0.94)	0.021	0.69 (0.59, 0.80)	< 0.001
Social independence						
Low	Ref.		Ref.		Ref.	
Medium	0.96 (0.89, 1.04)	0.324	1.00 (0.85, 1.18)	0.982	0.96 (0.89, 1.03)	0.257
High	0.87 (0.78, 0.97)	0.011	0.86 (0.68, 1.09)	0.208	0.88 (0.80, 0.98)	0.016
Decision-making						
Low	Ref.		Ref.		Ref.	
Medium	1.11 (0.98, 1.27)	0.110	1.12 (0.85, 1.48)	0.405	1.11 (0.97, 1.26)	0.120
High	1.12 (0.99, 1.28)	0.078	1.19 (0.91, 1.56)	0.205	1.10 (0.98, 1.25)	0.113

All models were adjusted for women age, employment status, number of children, wealth index, area of residence, and division

*APR* Adjusted prevalence ratio, *CI* Confidence interval, *Ref* Reference

## Discussion

To our knowledge, this is among the first studies to explore the association between women’s empowerment and several NCD-related conditions among WRA in Bangladesh. The majority of the women exhibited high empowerment in the attitude toward violence and decision-making domains, while a few showed social independence. A substantial prevalence of physical NCDs (overweight/obesity, hypertension, or diabetes), as well as symptoms of mental NCDs (anxiety or depression), was observed. Additionally, higher empowerment in the attitude toward violence domain was associated with lower prevalence of overweight/obesity and all forms of mental symptoms. High empowerment in social independence was also inversely associated with overweight/obesity, anxiety symptoms, and any mental symptoms. On the other hand, high decision-making empowerment was associated with a higher prevalence of overweight/obesity, hypertension, and any physical NCDs.

### Patterns of women’s empowerment

Consistent with earlier studies in Bangladesh [31, 40] and other South Asian countries [20, 41], this study found higher empowerment in the domains of decision-making and attitude toward violence, but lower empowerment in social independence. Women’s decision-making autonomy in Bangladesh increased from ~ 40% in 2011 to ~ 60% in 2022 [10, 42], while the proportion of women justifying wife-beating declined from 37% to 14% over the same period [10, 43]. These positive trends align with the high empowerment levels observed in our analysis. These improvements reflect the country’s progress in gender equality [44] and women’s participation in education and economic activities [45]. Legal frameworks such as the Prevention of Women and Children Repression Act, the Domestic Violence Act, the National Women Development Policy, and various programs led by the Ministry of Women and Children Affairs might have contributed to reducing violence and increasing women’s autonomy [46]. Nevertheless, progress in social independence remains limited. Many women continue to marry early, have restricted access to mass media, face adolescent childbirth, and a considerable proportion still lack formal education [45]. These persistent challenges highlight the need for continued policy and community-level interventions.

### Empowerment in the attitude toward violence domain and NCDs

This study demonstrated that women with high empowerment in the attitude toward violence domain were associated with lower prevalence of all forms of mental health symptoms, overweight/obesity, and the composite “any physical NCDs”, while no significant associations were found with hypertension or diabetes.

Intimate partner violence (IPV) is a major variable for this empowerment domain. The physiological pathways linking violence to these health outcomes are complex and include chronic activation of the stress response, dysregulation of the hypothalamic-pituitary-adrenal (HPA) axis, alterations in cortisol levels, increased allostatic load, inflammatory responses, and imbalances in neurotransmitters [47, 48]. These processes manifest more rapidly in mental symptoms through acute HPA-driven emotional dysregulation [47] and in overweight/obesity via cortisol-mediated appetite stimulation and visceral fat deposition [49]. A previous study in Bangladesh also linked the association between obesity and anxiety [50]. Hypertension and diabetes, by contrast, require prolonged exposure and additional genetic, behavioral, dietary, or metabolic factors to emerge clinically, explaining the null associations here. Furthermore, due to the high prevalence of overweight/obesity, the association in the composite outcome should be interpreted cautiously.

Sociocultural context is critical for such an association. In patriarchal settings such as Bangladesh, deeply entrenched cultural norms and unwritten social expectations often legitimize violence against women as an accepted component of family life [51]. Within this context, abusive behavior may be interpreted not as a violation of rights but as a justified expression of male authority, which women may internalize as being in their own best interest [52]. This normalization of violence can generate chronic psychological distress. Furthermore, IPV constrains women’s access to household resources, nutrition, healthcare, and financial autonomy, while stigma and low mental health literacy further impede care-seeking [53–55]. Conversely, women who reject violence may be less likely to internalize such norms and may experience greater autonomy, psychological resilience, and access to social and health resources, thereby reducing vulnerability to mental symptoms and adiposity.

These findings align with prior studies in Bangladesh [55–57], other LMICs [58, 59], and global analyses [60–62], linking IPV exposure to elevated mental disorders. However, evidence linking attitudes toward violence to physical NCDs remains mixed. An Indian study found an inverse association with hypertension [63], whereas research from Nepal observed null associations for hypertension [34] and mental health [35]. A systematic review further reported associations between IPV and diabetes and cardiovascular disease [64]. Furthermore, studies in LMICs found a positive association with IPV and overweight/obesity [65, 66]. Such divergence may reflect contextual heterogeneity in sociocultural norms, measurement of empowerment and IPV, variation in healthcare access, and diagnostic coverage across settings.

### Empowerment in the social independence domain and NCDs

Women’s empowerment in the social independence domain was inversely associated with overweight/obesity, anxiety symptoms, and the composite “any mental symptoms”. Furthermore, all other outcomes showed consistent inverse trends, though the association was not significant. Our findings align with prior evidence supporting a protective role of social independence on mental symptoms [35], but contrast with a study showing hypertension reductions in other settings [34].

Social independence—measured through education, access to information, age at first birth, and spousal differences—is critical for women’s autonomy and health behaviors. Women with higher social independence are more likely to delay marriage/childbirth, gain health knowledge, achieve elevated social status, seek healthcare proactively, and maintain social networks [14, 67]. These factors reduce stressor exposure and support healthier behaviors, plausibly underlying the significant inverse associations with mental health symptoms and overweight/obesity via improved stress management, dietary patterns, and physical activity. Non-significant yet directionally consistent trends for hypertension/diabetes likely reflect their longer latency. Overweight/obesity typically develops earlier as a direct consequence of behavioral and stress-related pathways, whereas prolonged obesity increases risk for these downstream cardiometabolic conditions, requiring extended exposure to manifest clinically [68, 69].

Despite these benefits, structural and sociocultural barriers in Bangladesh may attenuate social independence’s protective effects beyond observed outcomes. Constraints such as limited resources, economic opportunities, restricted mobility, physical activity, and persistent gender norms [70] could hinder translation of autonomy into broader health gains for non-significant outcomes. Multivariable adjustment for several variables may also represent over-adjustment, while a smaller sample size may mask true effects. This interplay underscores the need to address both individual empowerment and systemic barriers to optimize health benefits in Bangladesh and similar settings.

### Empowerment in the decision-making domain and NCDs

This study found a significant positive association between women’s empowerment in the decision-making domain and overweight/obesity, hypertension, and the composite physical NCD outcome, while no statistically significant associations were observed with mental symptoms. Similar positive associations with diabetes and anxiety were observed in unadjusted models. Evidence linking decision-making empowerment to physical NCDs remains limited and mixed. For instance, a study from Bangladesh reported higher odds of overweight/obesity among women with greater autonomy [71], whereas research from India found that women’s decision-making power had a protective effect against hypertension [72]. Furthermore, a study in Nepal found a null association with hypertension [34]. Regarding mental health, several studies from Africa [73–75] indicate a protective effect of decision-making empowerment, whereas some South Asian studies suggest an increase in mental health symptoms with greater empowerment [35, 76].

Bangladesh’s unique sociocultural context, particularly traditional gender norms, financial independence, and reliance on patriarchal approval, shapes health outcomes in ways that differ from other countries. Greater involvement in household decisions may increase role strain and psychosocial stress rather than alleviate it, particularly when empowerment is not accompanied by parallel gains in economic security or structural support [35, 77], potentially contributing to NCD risk. Furthermore, women with higher decision‑making autonomy may simultaneously gain control over health‑related choices and be more exposed to sedentary lifestyles, contributing to reduced energy expenditure and increased NCD risk. Empowered women might navigate physical healthcare more effectively, but mental health issues remain less acknowledged, possibly due to persistent stigma, cultural beliefs, and limited mental health literacy, which discourage women from recognizing or seeking formal care [78], limiting the observable associations between empowerment and mental health outcomes in this context.

These findings should be interpreted cautiously considering potential detection bias and temporal ambiguity. Women with greater decision-making power may have higher health awareness and better recognition of physical symptoms, leading to increased diagnosis and reporting of physical NCDs [79], which could produce higher observed prevalence through detection bias rather than higher underlying disease burden. Furthermore, the possibility of reverse causation could affect the interpretation, whereby existing NCDs alter household decision-making roles or increase engagement with health services, thereby inflating empowerment scores. Therefore, the observed association reflects a complex interplay of sociocultural context, behavioral pathways, and measurement limitations rather than a direct causal effect of decision-making empowerment on physical NCD risk.

### Policy implication

This study highlights the need to integrate women’s empowerment into public health strategies to mitigate the NCD burden in Bangladesh. To address gender-equitable attitudes toward violence, interventions should leverage existing platforms such as Community Clinics, Union Health and Family Welfare Centres (UHFWCs), and Upazila Health Complexes (UHCs), which already deliver maternal health, family planning, and basic services. These can incorporate community awareness campaigns, school-based education involving teachers, and advocacy through religious leaders and local government opinion leaders to promote mental health, reduce psychosocial stress, and facilitate early NCD detection. Enhancing social independence requires financial literacy and health education via community health workers, microfinance programs, and participatory workshops within reproductive health services. Given potential detection bias, where empowered women access healthcare more readily and receive earlier NCD diagnoses, empowerment efforts should pair with expanded NCD screening and management. This aligns with the government’s efforts to integrate mental health services into primary healthcare, including peer support groups and counseling in Community Clinics and maternal-child health programs. Policies targeting multiple empowerment dimensions alongside preventive NCD care would reduce health inequities and advance SDGs.

### Strengths and limitations

This study has several notable strengths. It utilized nationally representative data, ensuring the broad generalizability of the findings. The use of standardized survey methodologies and validated questionnaires enhanced the reliability and comparability of the data. Moreover, women’s empowerment was measured using the SWPER index—a globally recognized and validated tool—allowing for cross-country comparability and robust assessment across key empowerment domains. The inclusion of both physical and mental non-communicable conditions provides a more comprehensive understanding of how empowerment influences women’s health.

Despite these strengths, certain limitations should be acknowledged. The SWPER index, while widely used, does not fully capture the multidimensional nature of women’s empowerment, which also includes sociocultural, economic, legal, familial, political, and psychosocial dimensions. In addition, the analysis was restricted to ever-married women, limiting the generalizability of the findings to unmarried women. Mental health outcomes were assessed using the PHQ-9 and GAD-7 instruments, which are screening tools rather than diagnostic instruments. As a result, the prevalence of depression and anxiety symptoms may be overestimated or underestimated compared with clinically confirmed disorders. These outcomes were self-reported and may be subject to reporting and social desirability bias. Besides, the cross-sectional design precludes causal inference and raising the possibility of reverse causation. Women with existing mental or physical NCDs may experience changes in empowerment, household roles, or attitudes toward violence. In addition, greater interaction with health services among women with diagnosed conditions may influence both the reporting of empowerment-related variables and the likelihood of disease detection. Furthermore, residual confounding cannot be excluded, as several important factors were not available in the dataset, including dietary patterns, tobacco and alcohol use, physical activity, psychosocial stress, and social support. In addition, the selection of variables included in the adjustment models may have influenced the estimated associations. The analysis involved multiple comparisons across three empowerment domains and multiple NCD outcomes, increasing the possibility of type I error. Hence, these findings should be interpreted cautiously. Differences in sample size between mental health and physical NCD outcomes also limited statistical power for disease-specific analyses. A small number of observations were excluded due to missing data on key variables, which may introduce potential selection bias. Furthermore, the absence of a biomarker specific sampling weight in the BDHS dataset may slightly affect prevalence estimates. Longitudinal studies are needed to clarify temporality and causal pathways between women’s empowerment and NCD outcomes.

## Conclusions

Women’s empowerment influences the health outcomes of women in Bangladesh. Higher empowerment in the attitude toward violence was associated with lower prevalence of overweight/obesity and all forms of mental symptoms, while greater social independence was inversely associated with overweight/obesity, anxiety symptoms, and any mental symptoms. In contrast, higher decision-making empowerment showed positive associations with overweight/obesity, hypertension, and any physical NCDs—likely reflecting detection bias alongside lifestyle or socioeconomic mediators. These findings reveal complex pathways linking empowerment dimensions to health outcomes, underscoring the need for nuanced, domain-specific approaches in women’s health research and intervention design. Future studies should explore mediating mechanisms and longitudinal effects to inform targeted strategies for reducing Bangladesh’s growing NCD burden among women.

## Supplementary Information

Supplementary Material 1.

## Data Availability

All data are publicly accessible from the DHS database: https://www.dhsprogram.com/Countries/Country-Main.cfm?ctryid=1&c=Bangladesh&Country=Bangladesh&cn=&r=4.

## Abbreviations

AOR: Adjusted Odds Ratio
BDHS: Bangladesh Demographic and Health Survey
BMI: Body Mass Index
CI: Confidence Intervals
GAD-7: Generalized Anxiety Disorder-7
IPV: Intimate partner violence
LMICs: Low- and Middle-Income Countries
NCD: Non-Communicable Disease
PHQ-9: Patient Health Questionnaire-9
SDGs: Sustainable Development Goals
SWPER: Survey-based Women’s Empowerment
VIF: Variance Inflation Factor
WRA: women of reproductive age
