# Supplementary material for: Women’s empowerment and health: nationwide insights on selected non-communicable conditions in Bangladesh
Source: BMC Public Health. 2026 Mar 24;26:1168. doi: 10.1186/s12889-026-27084-y (PMC13064009; doi:10.1186/s12889-026-27084-y)
Supplement: Supplementary file 1 — Supplementary Material 1. [file 12889_2026_27084_MOESM1_ESM.docx]

**Supplementary Table 1.** Sociodemographic characteristics of the studied participants included in the analysis of hypertension/diabetes and any physical NCDs.

| **Variables** | **Hypertension/diabetes, N= 4493** | **Any physical NCDs, N= 4490** |
| --- | --- | --- |
|  | **n (%)** | **n (%)** |
| **Women age in years** |  |  |
| 15–29 | 1705 (37.8) | 1705 (37.9) |
| 30–39 | 1621 (36.5) | 1621 (36.5) |
| 40–49 | 1167 (25.7) | 1164 (25.6) |
| **Women education** |  |  |
| No education | 634 (14.2) | 633 (14.2) |
| Primary | 1204 (26.9) | 1203 (26.9) |
| Secondary | 1981 (45.4) | 1980 (45.4) |
| Higher | 674 (13.5) | 674 (13.5) |
| **Women employment status** |  |  |
| Not employed | 3060 (66.3) | 3057 (66.3) |
| Employed | 1433 (33.7) | 1433 (33.7) |
| **Number of children** |  |  |
| Up to 2 | 2934 (64.7) | 2933 (64.8) |
| 3 or more | 1559 (35.3) | 1557 (35.2) |
| **Wealth index** |  |  |
| Poorest | 796 (17.6) | 796 (17.6) |
| Poorer | 881 (20.5) | 881 (20.5) |
| Middle | 875 (20.0) | 874 (20.0) |
| Richer | 938 (21.1) | 938 (21.1) |
| Richest | 1003 (20.8) | 1001 (20.8) |
| **Area of living** |  |  |
| Urban | 1568 (28.0) | 1566 (28.0) |
| Rural | 2925 (72.0) | 2924 (72.0) |
| **Division** |  |  |
| Barishal | 516 (6.6) | 516 (6.6) |
| Chattogram | 672 (19.0) | 669 (18.9) |
| Dhaka | 618 (23.6) | 618 (23.6) |
| Khulna | 580 (12.1) | 580 (12.1) |
| Mymensingh | 495 (7.9) | 495 (7.9) |
| Rajshahi | 583 (13.4) | 583 (13.4) |
| Rangpur | 546 (11.5) | 546 (11.5) |
| Sylhet | 483 (5.9) | 483 (5.9) |
| **Standardized attitude toward violence SWPER score, mean (95%CI)** | 0.55 (0.53, 0.56) | 0.55 (0.53, 0.56) |
| **Standardized social independence SWPER score, mean (95%CI)** | −0.38 (−0.41, −0.35) | −0.38 (−0.41, −0.35) |
| **Standardized decision-making SWPER score, mean (95%CI)** | 0.38 (0.35, 0.41) | 0.38 (0.35, 0.41) |

**Supplementary Table 2.** Prevalence of anxiety symptoms, any mental symptoms and hypertension/diabetes by sociodemographic factors and SWPER domains.

| **Variables** | **Anxiety symptoms (GAD≥10)^1^, N= 18954** | | **Any mental symptom (GAD≥10 or PHQ≥10)^2^, N= 18954** | | **Hypertension/diabetes^3^, N= 4493** | |
| --- | --- | --- | --- | --- | --- | --- |
|  | **n (%)** | **p-value** | **n (%)** | **p-value** | **n (%)** | **p-value** |
| **Overall** | **777 (4.0)** |  | **1250 (6.4)** |  | **1209 (26.3)** |  |
| **Women age in years** |  |  |  |  |  |  |
| 15–29 | 238 (2.8) | <0.001 | 421 (4.9) | <0.001 | 216 (12.5) | <0.001 |
| 30–39 | 301 (4.7) |  | 463 (7.2) |  | 465 (28.1) |  |
| 40–49 | 238 (5.5) |  | 366 (8.4) |  | 528 (44.2) |  |
| **Women education** |  |  |  |  |  |  |
| No education | 140 (5.8) | <0.001 | 230 (9.4) | <0.001 | 212 (34.4) | <0.001 |
| Primary | 237 (4.6) |  | 359 (7.0) |  | 351 (28.9) |  |
| Secondary | 326 (3.8) |  | 522 (5.9) |  | 491 (23.8) |  |
| Higher | 74 (2.4) |  | 139 (4.4) |  | 155 (21.6) |  |
| **Women employment status** |  |  |  |  |  |  |
| Not employed | 525 (4.0) | 0.534 | 854 (6.4) | 0.527 | 824 (26.3) | 0.881 |
| Employed | 252 (4.2) |  | 396 (6.7) |  | 385 (26.5) |  |
| **Number of children** |  |  |  |  |  |  |
| Up to 2 | 454 (3.4) | <0.001 | 768 (5.6) | <0.001 | 727 (24.6) | 0.002 |
| 3 or more | 323 (5.6) |  | 482 (8.2) |  | 482 (29.4) |  |
| **Wealth index** |  |  |  |  |  |  |
| Poorest | 154 (4.6) | 0.181 | 244 (7.1) | 0.195 | 157 (19.4) | <0.001 |
| Poorer | 146 (3.9) |  | 250 (6.7) |  | 217 (23.5) |  |
| Middle | 159 (4.3) |  | 262 (6.8) |  | 224 (24.6) |  |
| Richer | 185 (4.4) |  | 265 (6.3) |  | 275 (29.2) |  |
| Richest | 133 (3.2) |  | 229 (5.4) |  | 336 (33.8) |  |
| **Area of living** |  |  |  |  |  |  |
| Urban | 256 (3.7) | 0.295 | 417 (5.9) | 0.227 | 510 (33.6) | <0.001 |
| Rural | 521 (4.2) |  | 833 (6.7) |  | 699 (23.5) |  |
| **Division** |  |  |  |  |  |  |
| Barishal | 72 (3.9) | 0.008 | 124 (6.4) | <0.001 | 126 (24.2) | 0.079 |
| Chattogram | 133 (4.7) |  | 178 (6.3) |  | 186 (26.8) |  |
| Dhaka | 99 (3.3) |  | 163 (5.5) |  | 178 (29.5) |  |
| Khulna | 138 (5.3) |  | 213 (8.1) |  | 152 (24.6) |  |
| Mymensingh | 60 (2.9) |  | 101 (5.0) |  | 117 (23.1) |  |
| Rajshahi | 63 (2.7) |  | 107 (4.5) |  | 151 (24.2) |  |
| Rangpur | 113 (5.2) |  | 212 (9.8) |  | 146 (24.6) |  |
| Sylhet | 99 (4.8) |  | 152 (7.4) |  | 153 (31.0) |  |
| **Attitude toward violence** |  |  |  |  |  |  |
| Low | 47 (6.7) | 0.002 | 75 (10.8) | <0.001 | 52 (29.3) | 0.239 |
| Medium | 95 (4.7) |  | 159 (7.8) |  | 134 (29.4) |  |
| High | 635 (3.8) |  | 1016 (6.1) |  | 1023 (25.8) |  |
| **Social independence** |  |  |  |  |  |  |
| Low | 378 (4.6) | 0.006 | 595 (7.3) | <0.001 | 548 (28.1) | 0.040 |
| Medium | 284 (3.7) |  | 459 (6.0) |  | 438 (24.2) |  |
| High | 115 (3.3) |  | 196 (5.2) |  | 223 (26.7) |  |
| **Decision-making** |  |  |  |  |  |  |
| Low | 83 (3.2) | 0.020 | 148 (5.6) | 0.162 | 147 (20.0) | 0.001 |
| Medium | 173 (3.6) |  | 302 (6.1) |  | 308 (26.2) |  |
| High | 521 (4.5) |  | 800 (6.8) |  | 754 (27.8) |  |

^1^Anxiety symptoms were defined as a GAD-7 score ≥10.

^2^Any mental symptom was a composite indicator defined as having either anxiety or depression symptoms; anxiety symptoms were defined as GAD-7 ≥10 and depression symptoms as PHQ-9 ≥10.

^3^Hypertension/diabetes was a composite indicator defined as having either hypertension or diabetes.

**Supplementary Table 3.** Unadjusted associations between women’s empowerment domains and different forms of physical NCDs.

| **Variables** | **Overweight/obesity** | | **Hypertension** | | **Diabetes** | | **Any physical NCDs** | |
| --- | --- | --- | --- | --- | --- | --- | --- | --- |
|  | **UPR (95%CI)** | **p-value** | **UPR (95%CI)** | **p-value** | **UPR (95%CI)** | **p-value** | **UPR (95%CI)** | **p-value** |
| **Attitude toward violence** |  |  |  |  |  |  |  |  |
| Low | Ref. |  | Ref. |  | Ref. |  | Ref. |  |
| Medium | 0.94 (0.83, 1.06) | 0.311 | 0.98 (0.67, 1.45) | 0.929 | 1.24 (0.77, 2.00) | 0.377 | 0.96 (0.84, 1.09) | 0.517 |
| High | 0.94 (0.85, 1.04) | 0.223 | 0.86 (0.62, 1.21) | 0.391 | 1.11 (0.71, 1.72) | 0.652 | 0.91 (0.81, 1.02) | 0.095 |
| **Social independence** |  |  |  |  |  |  |  |  |
| Low | Ref. |  | Ref. |  | Ref. |  | Ref. |  |
| Medium | 0.98 (0.93, 1.03) | 0.379 | 0.79 (0.68, 0.93) | 0.004 | 0.91 (0.76, 1.09) | 0.302 | 0.94 (0.9, 1.00) | 0.037 |
| High | 1.05 (0.99, 1.12) | 0.134 | 0.82 (0.66, 1.01) | 0.066 | 1.13 (0.90, 1.41) | 0.306 | 1.03 (0.97, 1.10) | 0.303 |
| **Decision-making** |  |  |  |  |  |  |  |  |
| Low | Ref. |  | Ref. |  | Ref. |  | Ref. |  |
| Medium | 1.30 (1.20, 1.40) | <0.001 | 1.27 (0.97, 1.68) | 0.087 | 1.45 (1.11, 1.90) | 0.007 | 1.16 (1.05, 1.27) | 0.002 |
| High | 1.37 (1.28, 1.47) | <0.001 | 1.53 (1.19, 1.97) | 0.001 | 1.35 (1.06, 1.72) | 0.016 | 1.21 (1.11, 1.31) | <0.001 |

UPR: Unadjusted prevalence ratio; CI: Confidence interval; Ref: Reference.

**Supplementary Table 4.** Unadjusted associations between women’s empowerment domains and different forms of mental symptoms.

| **Variables** | **Anxiety symptoms** | | **Depression symptoms** | | **Any mental symptoms** | |
| --- | --- | --- | --- | --- | --- | --- |
|  | **UPR (95%CI)** | **p-value** | **UPR (95%CI)** | **p-value** | **UPR (95%CI)** | **p-value** |
| **Attitude toward violence** |  |  |  |  |  |  |
| Low | Ref. |  | Ref. |  | Ref. |  |
| Medium | 0.83 (0.70, 0.99) | 0.040 | 0.86 (0.57, 1.31) | 0.492 | 0.87 (0.73, 1.03) | 0.102 |
| High | 0.65 (0.55, 0.76) | <0.001 | 0.62 (0.43, 0.90) | 0.011 | 0.66 (0.56, 0.77) | <0.001 |
| **Social independence** |  |  |  |  |  |  |
| Low | Ref. |  | Ref. |  | Ref. |  |
| Medium | 0.86 (0.80, 0.92) | <0.001 | 0.88 (0.74, 1.05) | 0.145 | 0.86 (0.80, 0.92) | <0.001 |
| High | 0.74 (0.67, 0.82) | <0.001 | 0.72 (0.57, 0.91) | 0.005 | 0.76 (0.69, 0.83) | <0.001 |
| **Decision-making** |  |  |  |  |  |  |
| Low | Ref. |  | Ref. |  | Ref. |  |
| Medium | 1.13 (0.98, 1.29) | 0.085 | 1.10 (0.84, 1.44) | 0.492 | 1.12 (0.98, 1.27) | 0.093 |
| High | 1.20 (1.06, 1.36) | 0.004 | 1.21 (0.93, 1.57) | 0.160 | 1.18 (1.05, 1.33) | 0.007 |

UPR: Unadjusted prevalence ratio; CI: Confidence interval; Ref: Reference.

**Supplementary Table 5.** Associations between women’s empowerment domains and composite diabetes/hypertension.

| **Variables** | **Diabetes/hypertension** | | **Diabetes/hypertension** | |
| --- | --- | --- | --- | --- |
|  | **UPR (95%CI)** | **p-value** | **APR (95%CI)^1^** | **p-value** |
| **Attitude toward violence** |  |  |  |  |
| Low | Ref. |  | Ref. |  |
| Medium | 1.00 (0.75, 1.34) | 0.976 | 0.99 (0.75, 1.30) | 0.941 |
| High | 0.88 (0.68, 1.14) | 0.339 | 0.87 (0.68, 1.11) | 0.263 |
| **Social independence** |  |  |  |  |
| Low | Ref. |  | Ref. |  |
| Medium | 0.86 (0.77, 0.97) | 0.015 | 0.94 (0.84, 1.06) | 0.338 |
| High | 0.95 (0.82, 1.09) | 0.472 | 0.88 (0.76, 1.01) | 0.073 |
| **Decision-making** |  |  |  |  |
| Low | Ref. |  | Ref. |  |
| Medium | 1.31 (1.08, 1.60) | 0.008 | 1.21 (1.00, 1.46) | 0.045 |
| High | 1.39 (1.16, 1.67) | <0.001 | 1.18 (0.99, 1.41) | 0.058 |

APR: Adjusted prevalence ratio; UPR: Unadjusted prevalence ratio; CI: Confidence interval; Ref: Reference.

^1^ All models were adjusted for women age, employment status, number of children, wealth index, area of residence, and division.

**Supplementary Table 6.** Associations between women’s empowerment domains and anxiety (using GAD≥10 cut point) and any mental symptoms.

| **Variables** | **Anxiety symptoms (GAD-7≥10)** ^1^ | | **Anxiety symptoms (GAD-7≥10)** ^1^ | | **Any mental symptoms (GAD-7≥10 or PHQ-9≥10)** ^2^ | | **Any mental symptoms (GAD-7≥10 or PHQ-9≥10)** ^2^ | |
| --- | --- | --- | --- | --- | --- | --- | --- | --- |
|  | **UPR (95%CI)** | **p-value** | **APR (95%CI)** ^3^ | **p-value** | **UPR (95%CI)** | **p-value** | **APR (95%CI)** ^3^ | **p-value** |
| **Attitude toward violence** |  |  |  |  |  |  |  |  |
| Low | Ref. |  | Ref. |  | Ref. |  | Ref. |  |
| Medium | 0.70 (0.47, 1.06) | 0.091 | 0.71 (0.47, 1.06) | 0.097 | 0.72 (0.52, 1.01) | 0.054 | 0.72 (0.52, 1.01) | 0.054 |
| High | 0.57 (0.41, 0.80) | 0.001 | 0.60 (0.43, 0.84) | 0.003 | 0.56 (0.43, 0.74) | <0.001 | 0.58 (0.44, 0.76) | <0.001 |
| **Social independence** |  |  |  |  |  |  |  |  |
| Low | Ref. |  | Ref. |  | Ref. |  | Ref. |  |
| Medium | 0.80 (0.66, 0.97) | 0.021 | 0.92 (0.76, 1.11) | 0.369 | 0.82 (0.71, 0.95) | 0.009 | 0.93 (0.80, 1.07) | 0.294 |
| High | 0.70 (0.55, 0.90) | 0.005 | 0.85 (0.65, 1.10) | 0.215 | 0.71 (0.59, 0.86) | <0.001 | 0.83 (0.68, 1.01) | 0.062 |
| **Decision-making** |  |  |  |  |  |  |  |  |
| Low | Ref. |  | Ref. |  | Ref. |  | Ref. |  |
| Medium | 1.12 (0.83, 1.52) | 0.463 | 1.13 (0.84, 1.52) | 0.431 | 1.09 (0.86, 1.37) | 0.480 | 1.09 (0.87, 1.37) | 0.461 |
| High | 1.41 (1.04, 1.93) | 0.029 | 1.33 (0.96, 1.83) | 0.083 | 1.21 (0.96, 1.51) | 0.101 | 1.15 (0.91, 1.46) | 0.234 |

APR: Adjusted prevalence ratio; UPR: Unadjusted prevalence ratio; CI: Confidence interval; Ref: Reference.

^1^Anxiety symptoms were defined as a GAD-7 score ≥10.

^2^Any mental symptom was a composite indicator defined as having either anxiety or depression symptoms; anxiety symptoms were defined as GAD-7 ≥10 and depression symptoms as PHQ-9 ≥10.

^3^ All models were adjusted for women age, employment status, number of children, wealth index, area of residence, and division.
